# Immersive learning in medical education: analyzing behavioral insights to shape the future of VR-based courses

**DOI:** 10.1186/s12909-024-06337-7

**Published:** 2024-12-03

**Authors:** Anna Junga, Henriette Schulze, Sönke Scherzer, Ole Hätscher, Philipp Bozdere, Paul Schmidle, Benjamin Risse, Bernhard Marschall, the medical tr.AI.ning consortium

**Affiliations:** 1 Institute of Education and Student Affairs, University of Münster, Niels-Stensen-Straße 12, Münster, 48149 Germany; 2 Department of Psychology, University of Münster, Münster, Germany; 3 Institute for Society and Digital Media, Münster University of Applied Sciences, Münster, Germany; 4 Department of Dermatology, Medical Faculty, University of Münster, Münster, Germany; 5 Institute for Geoinformatics, University of Münster, Münster, Germany; 6 Faculty of Mathematics and Computer Science, University of Münster, Münster, Germany

**Keywords:** Computer simulation, Education, Medical, Immersion, Hygiene, Virtual reality, Simulation training, Survey method

## Abstract

**Background:**

The emergence of virtual reality (VR) for medical education enables a range of new teaching opportunities. Skills and competences can be trained that cannot be demonstrated in any other way due to physical or ethical limitations. Immersion and presence may play an important role for learning in this context. This study investigates whether this VR-based, immersive software is an effective tool for assessing medical learning objectives by comparing behavioral outcomes in VR and actor-based simulations, and examines how these behaviors relate to immersion levels and their impact on learning success.

**Methods:**

To evaluate the effectiveness of the new teaching method, objective behavioral outcomes were identified as part of a dermatological learning unit and VR as a method was compared with actor-based simulation training. In addition, subjective questionnaires were collected to compare the levels of immersion in both concepts.

**Results:**

It was shown that primary learning objectives can be addressed well in VR. However, secondary learning objectives that fall into the field of basic skills seem to be delivered better in the actor-based training than in VR. This appears to be an effect of weaker immersion measured in VR training.

**Conclusions:**

It can be said that the implementation of basic skills training depends largely on the level of immersion in the teaching method used. While primary learning subjectives can be trained and assessed well, at present, it does not appear to be fully possible to train secondary skills with the technical status quo in VR. However, the observation of secondary learning objectives can serve as an indicator for the assessment of immersion in the future.

**Supplementary Information:**

The online version contains supplementary material available at 10.1186/s12909-024-06337-7.

## Background

Major technological advances in recent years, such as increasingly high-resolution imaging and software solutions, as well as the COVID pandemic as an additional catalyst [1] have made virtual, augmented, and mixed reality (VR, AR, and MR) applications increasingly popular [2, 3]. This also applies to the medical sector: Besides conventional 2D applications such as laparoscopy trainers and their 3D developments (e.g. DaVinci robot system trainers) [4, 5], applications in the field of artificial intelligence-based interaction are currently on the rise. These techniques have not only revolutionized preoperative planning [3, 6–8] but also postoperative/general care [2, 9, 10].

In the field of medical education, VR is playing an increasingly important role e.g. in residency training for physicians (e.g. disaster training [11, 12], cardiopulmonary resuscitation [13], craniofacial trauma [14] and training and education of nurses [15–17]). However, VR has not yet been widely adopted in traditional student education and simulation training [18]. This may partly be due to the limitations of VR technology, including cost-effectiveness but also the lack of statistical evidence of its educational use under real conditions [19].

However, the use of actors as simulated patients or manikins is widely accepted in the education of medical students and has been part of the teaching routine in numerous medical faculties for many years. Nevertheless, these simulations have several limitations that VR can address. While VR cases offer the same safe environment and situated learning opportunity as other simulation trainings, they can also showcase medical areas that are often excluded from the curriculum. Barriers such as patient safety or privacy, rare diseases or patients and conditions that can't be represented by manikins or actors can be overcome with the use of VR [20]. Examples for VR trainings that address those issues are a brain death examination [21, 22], a dermatological full body skin cancer examination [23, 24], post-mortem examinations [25], operative stray radiation safety training [26] or pediatric emergencies training [27]. Also, VR emergency trainings are on the rise that address multiplayer modes and are built on complex physiological engines, that match treatments realistically in real time [28–31]. Additionally, a comprehensive review of the use of VR in medical education can be found in [32].

Another benefit of VR is its visually realistic imaging, which is currently predominantly realized by Head-Mounted-Display (HMD)-based systems. It has been shown that high levels of immersion can be achieved with the help of HMDs [33]. Immersion is often described as the technical ability of a system to present an intense virtual environment while shutting out physical reality [34, 35]. While experts in the field of media studies largely agree on immersion being an important phenomenon when interacting with any kind of media (even two-dimensional) [36], VR is especially suited to draw users into the depicted world. The ability to flood multiple of the users´ sensory channels with artificial stimuli while drowning out others leads to a high degree of immersion in the depicted situation [37]. The application of VR aims to create an intense immersive experience with the help of 3D visual, hapto-tactile and acoustic impressions, that reacts upon movement and interaction.

If the VR system possesses a high level of immersion, it can make users feel as if they were actually “there” – a phenomenon called “presence”, the subjective correlate of immersion [34, 38]. This allows the virtual experience to evoke both the physical and emotional aspects of a natural habitus. Considering this phenomenon, it is not surprising that VR can be used to induce not only feelings of joy, but also negative emotions such as stress, anxiety, and disgust. If VR is used correctly, it can even induce intense physical reactions although the user is performing in a safe environment (e.g. exposure therapy in psychotherapy [39]). Within the same mechanism, VR technology can be used to create complex situations to train not only knowledge or motor skills (e.g. surgical suture training) but also complex emotional and interpersonal competencies.

One major question in this regard is which degree of immersion is needed to enable learning success. It has been suggested that learning is especially successful, if it occurs in an environment close to reality and enhances an active construction of knowledge, rather than passively absorbing it in the form of lectures, for example [40]. This so-called situated learning is an essential basis for learning during simulations and therefore also VR-based training. It recently has been discussed how learning is influenced by immersion. A theory concludes that the immersion of HMD-based VR trainings leads to high levels of presence and agency, which in return may enhance psychological factors associated with learning, like interest, self-motivation, self-efficacy and more [41]. Nevertheless, empirical results testing this theory are mixed and seem to depend on the learning objectives [42].

Additionally, former studies mainly focused on the effect of immersion on a specific learning objective in different domains [42]. What has not been thoroughly investigated is whether the level of immersion influences direct learning objectives in the same way it influences indirect learning objectives. For this study, we focused on primary learning objectives (those that were asked for in the task e.g. performing a correct surgical suture) and secondary learning objectives, which are not mentioned in the task but are routine actions that should be consolidated in further training (e.g. hand hygiene or sterile dressing of surgical gowns if necessary).

Another important aspect is how immersion is currently measured. Traditionally, measurements of immersion are based on subjective post-event surveys of students where they can rate various aspects of how realistic the scenario appeared to them. This has the drawback that the measurement relies on the participants recalling their experience. Furthermore, it is unclear how adequate participants can judge implicit processes, such as embodiment and presence, through post questionnaire ratings [43, 44]. Thus, additional methods of measuring immersion would be beneficial to get a more comprehensive understanding of the immersion of simulated environments. A novel approach in medical education context is to study the behaviour of the participants in the simulation: If the participants´ behaviour is close to a real-world situation, this could indicate a high degree of immersion of the simulated environment.

An application was developed in which medical students are able to autonomously perform a dermatological examination on a digital patient in VR. This software was then integrated into the medical curriculum at Münster University and Saarland University.

This study aims for two main objectives: At first, we wanted to evaluate if the analyzed VR-Software, as an example of a VR-based (immersive and HMD bound) competence-based assessment, is an effective vehicle to test medical learning objectives. The behavioral outcomes of primary and secondary learning objectives were examined separately for a VR- and actor-based simulation. Secondly, behavioral differences were compared to post-rated presence questionnaires (to analyze the level of immersion). It will be discussed to what extent behavioral differences can be used to derive the level of immersion and how this evaluation may predict the suitability of different learning objectives in medical VR-based trainings.

## Methods

The Medical Faculty of the University of Münster is home to a highly modern teaching facility for students (LIMETTE training center). This institution offers medical students hands-on training through engagement with professional actors who simulate patient scenarios, all within a controlled environment. Supervised training is conducted across a variety of scenarios, offering a comprehensive learning experience. At Münster University, in cooperation with Münster University of Applied Sciences, Saarland University and Saar University of Fine Arts, a VR-application (‘Skin cancer screening’ within the funded research project’medical Tr.AI.ning’ [23, 24]) was developed and implemented for the first time in the summer term of 2023 in which students were tasked with performing a virtual full-body skin cancer screening as a simulation competency training. This course was developed for the Valve Index Head-Mounted Display (HMD) on basis of the Unity Game Engine (Unity Technologies, San Fransisco, USA, version 2021.3), further technical details can be found in [24].

### Learning objectives

To evaluate the dermatological VR application, surveys were conducted among students regarding usability, presence and other factors. At the same time, the participating students were recorded while performing their tasks in VR. This was compared to students of the same academic year, who were observed during a traditional actor-based dermatology simulation training in the training center ‘LIMETTE’. All participants were asked for permission to use the recorded video data on a voluntary basis.

Firstly, recognizing and documenting conspicuous skin lesions was defined as a measure of the primary learning objectives. Further behavioral items were identified that act as a basic building block of ethical and routine hygienic medical action, which can be seen as mandatory in direct patient contact, but also as secondary learning objectives for every suitable simulation training case.

These items were:

1. “closing the examination room´s door” for data protection and to protect the patient´s privacy, especially while performing obligatory intimate examinations
2. “using hand disinfection before and after patient contact” to protect the patient from transmission of pathogens via the examinator´s hands
3. “using medical gloves” for self-protection, to avoid skin contact with e.g. infectious material or parasites

According to the NKLM (German “National Competence Based Learning Objectives Catalogue Medicine”), the listed competences should be able to be applied fully independently and adequately by students from 7 to 10th semester at the latest (chapter VIII.4–04.1.1; VIII.7–01.1.1 and VIII.6–01.2.8 [45]). The 10th semester was selected for the survey. The competencies mentioned are therefore essential requirements for students in the 10th semester, immediately before taking the theoretical state examination in Germany. Clear deviations from this are conspicuous and could be an indication of an inability to comply with basic requirements.

**Fig. 1:**
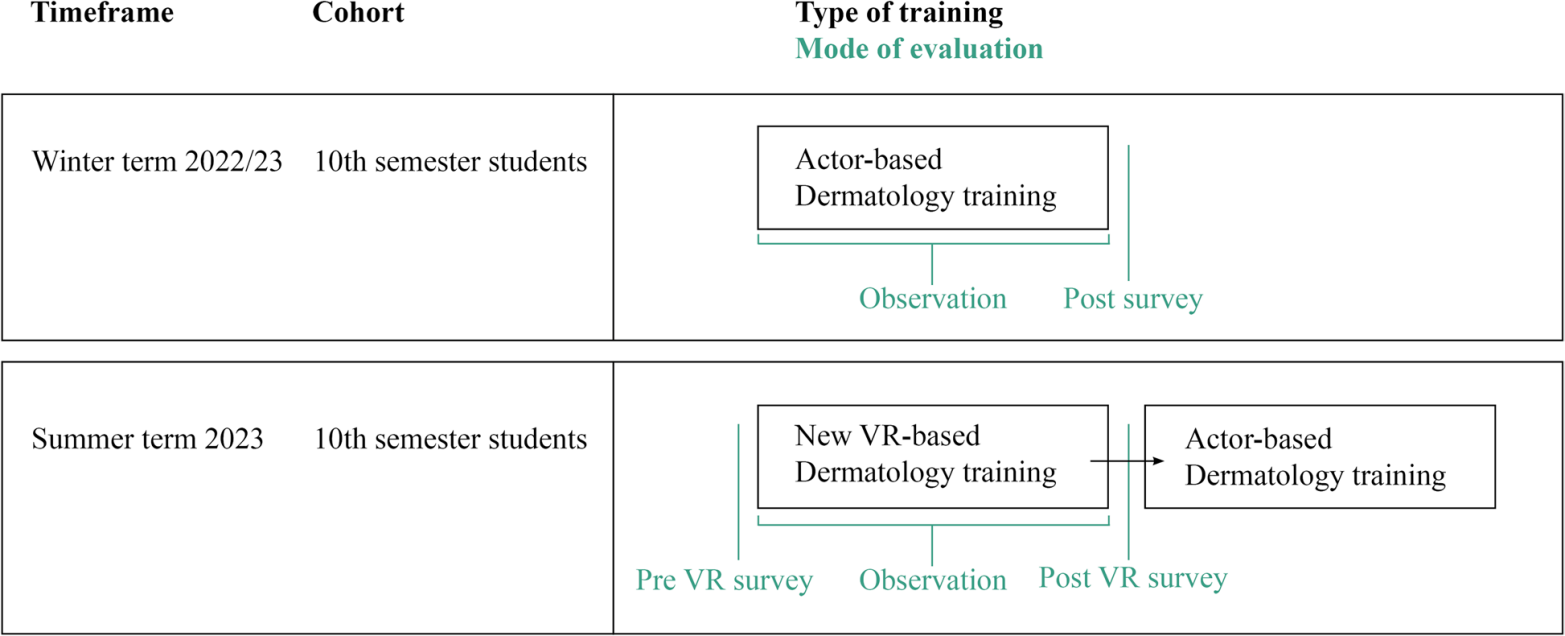
Schematic representation of the study design. Two cohorts were used in order to avoid distortion of the data by the other course module

In order to establish a baseline, the students' behavior was first observed and evaluated in a real, actor-based course. This control group is from the 10th semester of the winter term 2022/2023 while the test group is from 10th semester of summer term 2023. The VR group was additionally asked to fill in questionnaires before, during and after the course for a more detailed evaluation of the new course format, which was conducted for the first time in this winter term (see Fig. 1). Included were demographic data, the System Usability Scale (SUS) to determine usability [46] and the Reality Judgement and Presence Questionnaire (RJPQ) [47]. VR in-game recordings were used to observe the same behavioral aspects than in the control group.

### Simulation scenarios

**Fig. 2:**
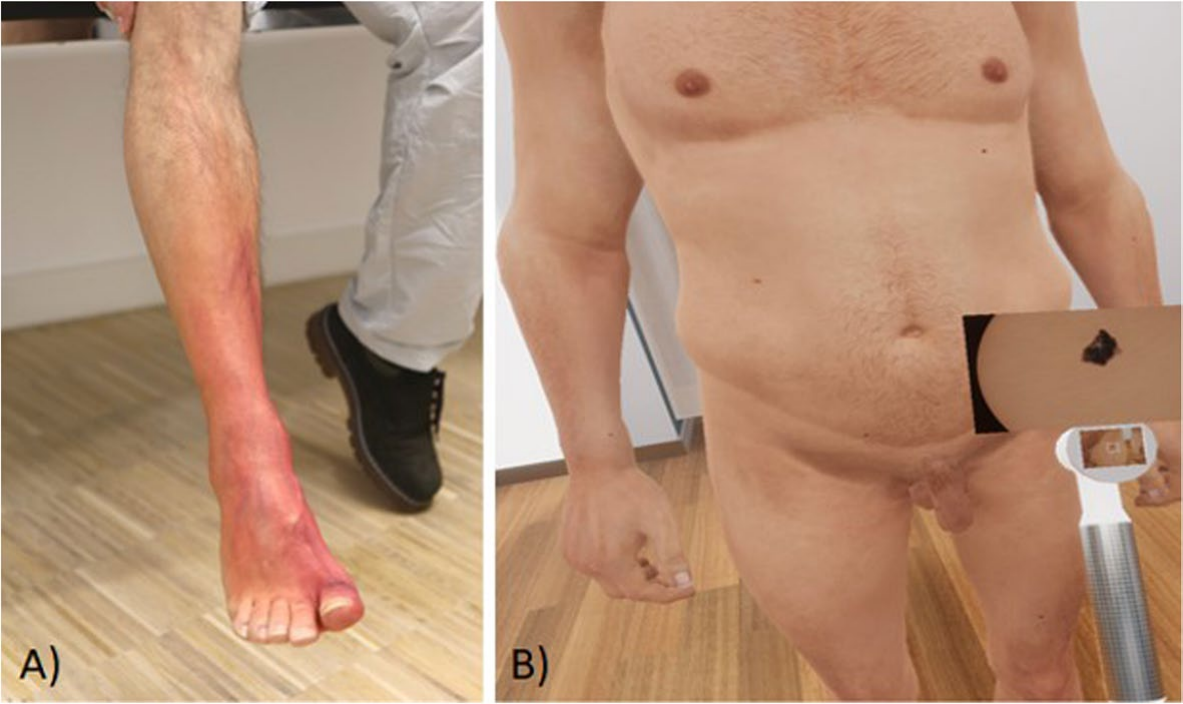
Illustration of the two pathologies to be examined as part of the courses. **A** Actor-based course: Right leg of the simulation patient with erysipelas. A clear reddening can be seen starting from the big toe (overheating also palpable). **B** Virtual-reality based course: Partial section of the undressed virtual patient with melanoma of the left inguinal area (scenario 1 of 5), which is examined using a dermatoscope

The task of the reference group is part of a multi-level training parcourse of the interaction training for dermatology within the 10th semester. Participation is obligatory for every student. The students were given the task of examining a patient who presents a painful erythematous leg (see Fig. 2A) and an itchy spot on the back. The direct manual examination is mandatory to detect important symptoms like overheating and palpatory findings. The suitability of this teaching concept for achieving the primary learning objectives has already been evaluated and optimized several times in internal evaluations over the last few years and is therefore regarded as appropriate. Every student was alone in a moc-examination room with one middle-aged Caucasian male actor. The time limit for the task was 10 min, and the students had the task to perform medical history and a symptom-related examination (see Appendix 1). No additional materials were provided other than the standard room equipment which consisted of medical gloves and hand disinfectant. A stethoscope was brought by the students. The scene was observed by a teacher that was connected via an intercom system and could not be seen from the room. No content-related help was given.

**Fig. 3:**
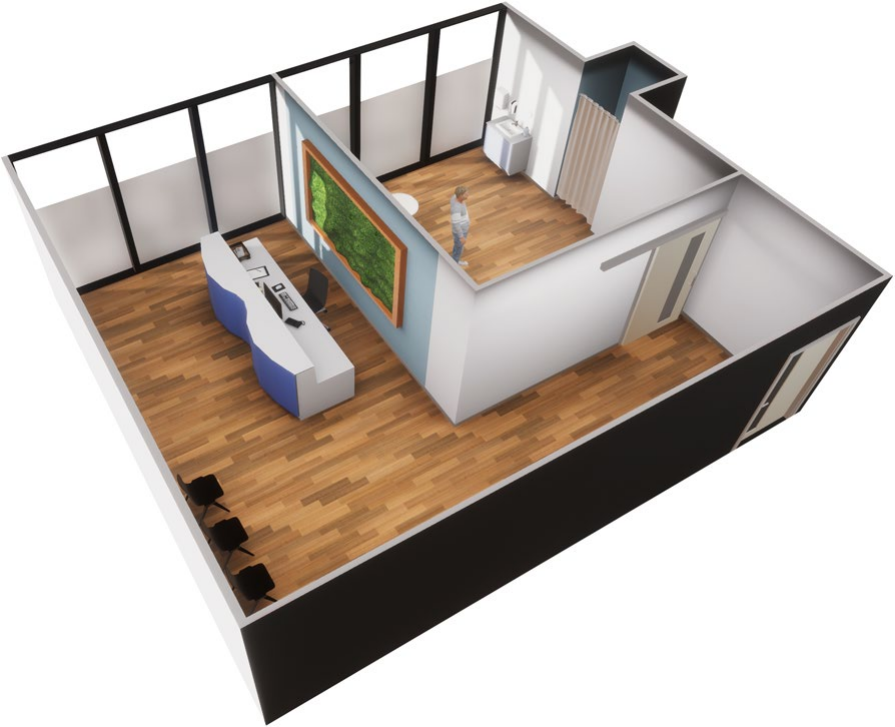
Aerial view of the dermatological practice with the waiting room on the left and the examination room with changing room on the top left (generated graphic, no in-game image)

**Fig. 4:**
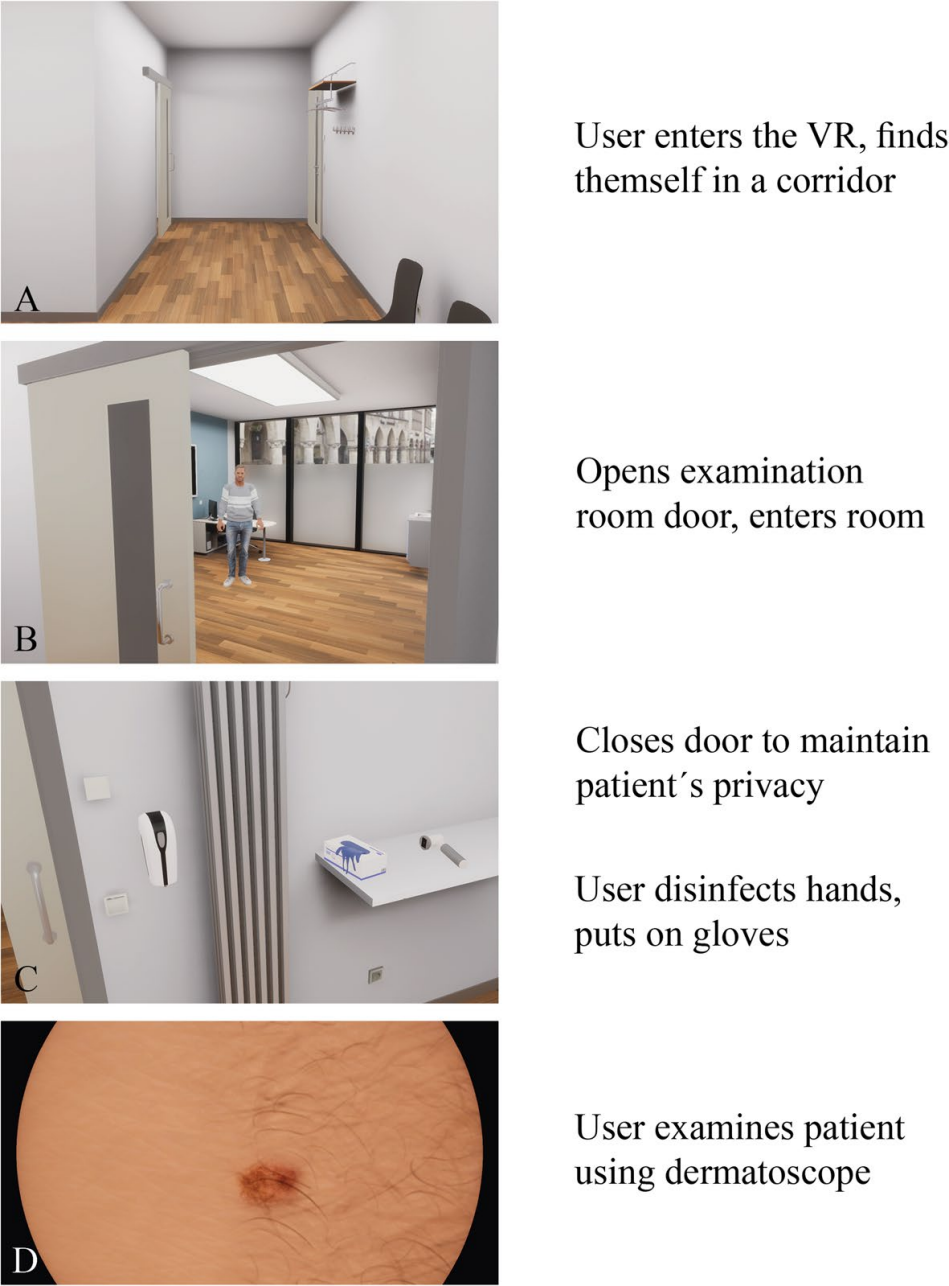
Schematic sequence of steps within the software (user point-of-view): Image **A** shows the waiting room, where the application starts. The student must navigate independently to the examination room. **B** After opening the door, the examination room can be entered. The patient is waiting there and can be interacted with via a dialogue menu. **C** shows the available utensils (from left to right: disinfectant dispenser, medical gloves, dermatoscope). **D** Visualization of the skin under the dermatoscope

The VR course group (also 10th semester) had a theoretical and practical introduction in VR hard- and software by a standardized tutorial beforehand in order to create equal conditions for all students. This preparation includes moving in virtual rooms so as interaction with objects and using the dialogue menu without spoiling any information. The primary learning objective was to conduct an examination for skin abnormalities, including taking a medical history of an unknown patient in a virtual practice (see Figs. 2B, 3 and Appendix 2). The results were discussed with a teacher afterwards. Like the actor-based course design, the students worked in the same real rooms on their own with one hardware setup and one (virtual, middle-aged Caucasian) patient each. Due to the slightly different task, the students in the virtual room had a dermatoscope to use in addition to medical gloves and disinfectant. This was used to record the anomalies, which were then discussed in the following (non virtual) debriefing. It was also possible to take a specific medical history via dialogue system and to perform a specific body examination. The patient could be asked to assume various poses via the dialogue menu to make all parts of the skin visible (e.g. bending forward, standing on one leg, stretching and rotating arms, etc.). Undressing and obtaining the patient's consent could also be ca rried out via the dialogue menu (see Figs. 3 and 4). The observation was conducted within the same system supplemented with a screen mirroring system to see the VR-view every time. No content-related assistance or feedback was given within the simulation, only technical advice was given if needed. The time limit was 25 min to complete the task.

In both cases, the patient has to be undressed (partly or fully) and hygiene standards must be maintained. Closing the door is mandatory in both setups to protect the patients’ privacy and to ensure data protection. As the actor-based training took place two weeks after the VR training and the hygiene standards were an explicit part of the VR debriefing, we decided to compare two different cohorts to avoid a priming effect. The students evaluated in the actor-based course did not perform the VR course before and vice versa.

As the comparison group did not perform in reality, but in a realistic form of simulation, the RJPQ (adapted formulation) was also queried in the actor-based course format to obtain a baseline. Two-sided t-tests for independent samples were performed to identify significant differences in the RJPQ dimensions between the two groups.

To analyze the behavioral data, the videos were inspected. Various measurement points were collected, partly in binary values, partly in time stamps, and mean values were determined. Table 1 shows all measurement points. Multiple Chi^2^-Tests (χ2) were performed to compare the binary behavioral variables (door closed, used hand disinfection, used medical gloves) between both groups.

**Table 1:** Monitored objectives within the VR and actor-based simulations, valid values and unit of measurement

Action	
door opened timecode (time baseline)	timecode; mm:ss
door closed	0 = no; 1 = yes
door closed timecode	timecode; mm:ss
door closed before undressing the patient	0 = no; 1 = yes
used hand disinfection	0 = no; 1 = yes
used hand disinfection first time	timecode; mm:ss
used hand disinfection before patient contact	0 = no; 1 = yes
used hand disinfection after patient contact	0 = no; 1 = yes
used medical gloves	0 = no; 1 = yes
used medical gloves before first examination contact	0 = no; 1 = yes
put gloves on	timecode; mm:ss
number of used gloves	value
removed gloves before leaving the room	0 = no; 1 = yes
duration of simulation	timecode; mm:ss

## Results

### General evaluations

In the VR group, 95 of 140 students agreed to participate in the extended evaluation of the course. 85 videos were available to conduct the extended analysis. In the actor-based group 30 out of 66 students provided their video data for the analysis.

The majority of students in VR (81.8%) reported that they have already used a VR headset once or rarely. However, only 1.8% reported using a VR headset on a regular basis.

Within the VR group survey there were 69.8% female students (0% diverse). The mean age of the respondents was 25.4 years. This corresponds to the proportion of women in this field of study and the median age of medical students in this stage of their studies [48, 49]. Since both groups (VR- and actor-based) are from the 10th semester at the same university in the same study program (with a difference in study participation of 6 months), it can be assumed that the study groups are comparable.

The examination time differed between the two course groups. Due to course structures the real training group had 10 min, with a reminder after 8 min that time will be up soon. The VR group had about 25 min with a reminder after 20 min. In general, students need additional time in VR because the technical handling is different than normal (e.g. use of controllers for interactions). The average time needed for the actor-based group was 7:43 min (± 01:03 min), for the VR group 18:45 min (± 05:00 min).

### Primary learning objectives and immersion

In VR simulation all students performed an anamneses and 74,22% of the students managed to identify at least one (of one or two melanoma placed) lesion correctly (primary learning objective). The students were furthermore asked about their subjective learning success at various points during the VR course. The subjective learning success showed a constant increase with the greatest increase after the VR application itself (t_0_ = 2.09 ± 0.85; t_1_ = 2.28 ± 0.87; t_2_ = 3.12 ± 0.9; t_3_ = 3.34 ± 0.81; Δt_0–1_ = 0.19 vs. Δt_1–2_ = 0.84 vs. Δt_2–3_ = 0.22).

**Fig. 5:**
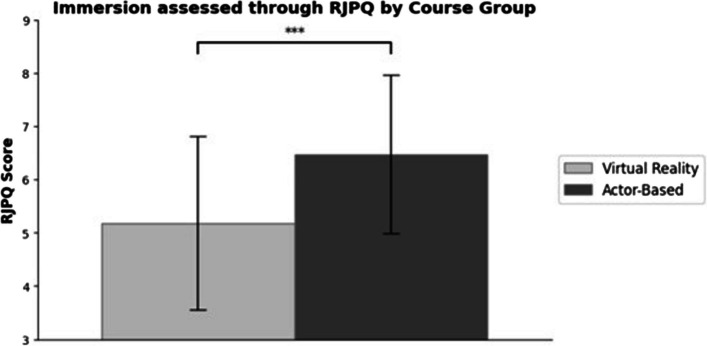
This figure shows the difference in the Immersion obtained through the RJPQ between the VR-based course (light grey) and the actor-based course (dark grey); mean ± SE; *n* = 90 (VR) and *n* = 66 (actor). ***p < 0.001, scale adapted for better visualization

The Reality Judgment and Presence (RJPQ) Questionnaire was used within the VR course to evaluate students' perceptions of immersion and realism (min. 0, max. 10) (see Fig. 5). The mean value for attention/absorption was 5.76 ± 1.95, for internal/external correspondence 5.27 ± 1.70 and for reality judgment 4.82 ± 2.01. The actor-based reference group reported 6.46 ± 1.77 for attention/absorption (Δ VR *p* = < 0.05), 6.11 ± 1.58 for internal/external correspondence (Δ VR *p* = < 0.01) and 6.76 ± 1.79 for reality judgment (Δ VR *p* = < 0.001).

### Secondary learning objectives

In order to classify the results, secondary learning objectives were monitored in the VR simulation. To determine the current state of proficiency, the same procedures were also observed in the comparison group.

In the actor-based group 100% of the participants closed the door immediately (4 s ± 1 s) after opening the door and therefore before patient contact. In the VR group only 15% closed the door, on average 57 s (± 49 s) after opening it. In the few cases the door was closed, it happened at least before undressing the patient. Thus, there was a significant difference between the two groups (χ2[1, *N* = 115] = 64.4, *p* < 0.001). The second aspect investigated whether gloves were worn before examining the potentially infected legs. In the actor-based group 97% used gloves overall, these were (100%) put on before first skin contact. As in this case the patient presented with two skin abnormalities a few students changed gloves during the examination, the mean glove consumption was 2.43 gloves per student [100 available]. 100% of the students took the gloves off before leaving the examination room. In the VR group 49% of the students used gloves (mean 0,9 gloves per student [max. 2]), but only 39% did this before first skin contact. At that time, there was no functionality in the software to take the gloves off again. The proportion of students who used gloves at any time differs significantly between the two groups (χ2[1, *N* = 115] = 18.2, *p* < 0.001).

The last item observed was the correct usage of hand disinfection. In the actor-based group 93% used hand disinfection in general, 67% before the first skin contact. 83% of the students used hand disinfection after the last skin contact with the patient. A closer look at the timeline reveals two groups: Group 1 disinfected their hands immediately after entering the room (after 07,00 s ± 04,50 s), group two did so directly before the physical examination (after 304 s ± 159 s). In the VR-based group 21% used hand disinfection, 13% before the first skin contact and only 8% after the last skin contact (χ2[1, *N* = 115] = 45.1, *p* < 0.001). The two behavioral groups "disinfection before the anamnesis interview" (78,00 s ± 45,50 s) and "disinfection immediately before the physical examination" (970 s ± 190,10 s) can also be identified here.

**Fig. 6:**
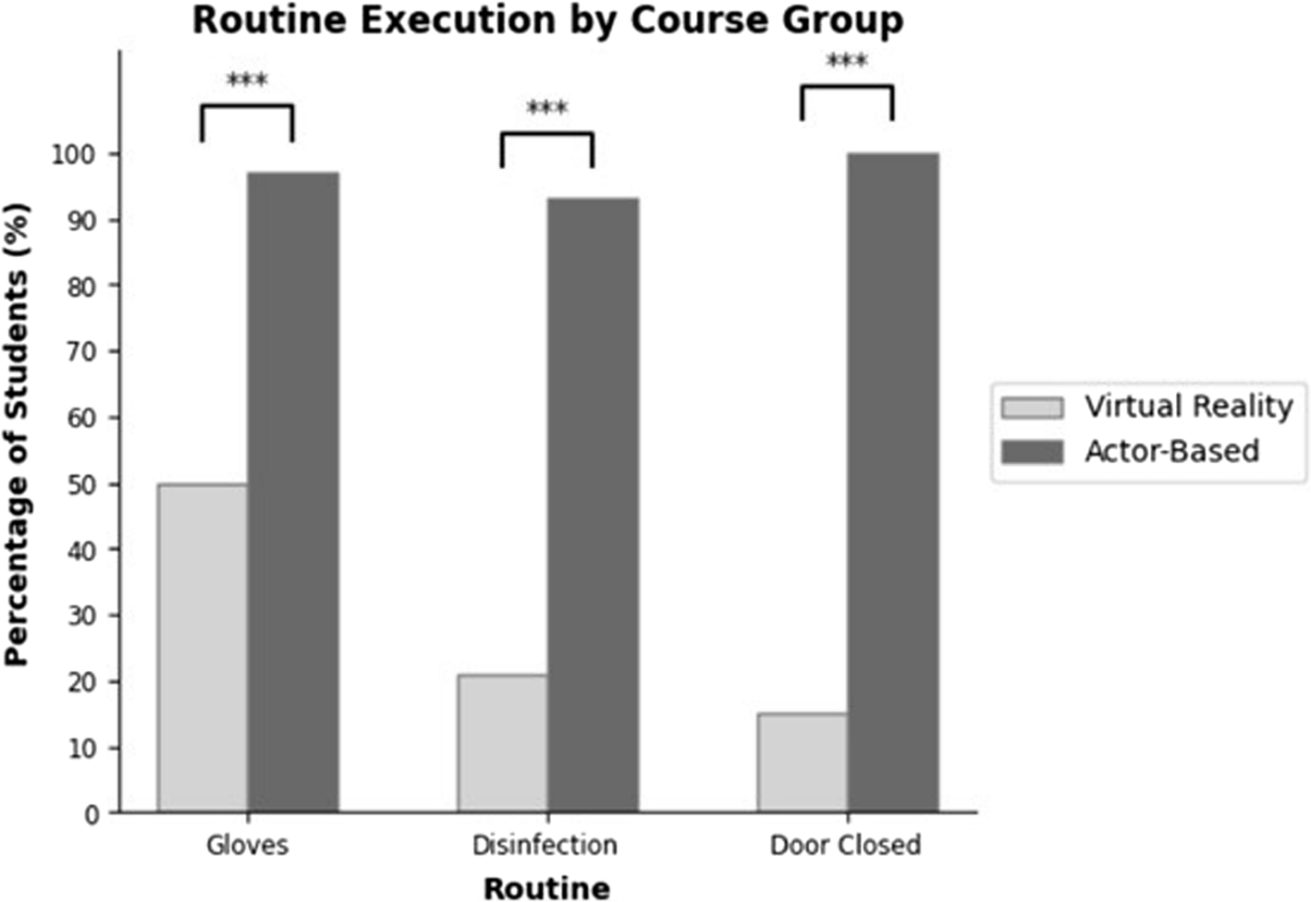
This figure shows the difference in the routine execution frequency between the VR-based course (light grey) and the actor-based course (dark grey). (*n*=85 (VR) and *n*=30 (actor)). ***p < 0.001

The differences in the execution of medical routines between the two groups are visualized in Fig. 6.

Additionally, the usability was measured via SUS in the VR group. The mean value was 70.92 ± 15.53 (min. 0, max. 100), which can be interpreted as medium-good results. Just under a third (28%) of the students rated the usability between 80% (good) and 100% (very good). Further analysis showed that there is a correlation between previous VR experiences and the SUS score (τ = 0.26, p < 0.01). The statistical comparisons between the two groups in secondary learning objective and immersion are displayed in Table 2. It shows that the scenario was perceived as significantly more immersive in the actor-based group than in the VR group. Furthermore, the secondary learning objectives were significantly better achieved in the actor-based group.

**Table 2:** Statistical comparisons in immersion and routine execution between the two groups

Variable	M (SD) / H VR group	M (SD) / H Actor-based group	Statistical test	Test statistic	*p* value
**RJPQ**					
Attention / absorption	5.76 (1.95)	6.46 (1.77)	t-test	t = −2.31	0.02
Int. / ext. correspondence	5.27 (1.70)	6.11 (1.58)	t-test	t = −6.23	< 0.001
Reality judgement	4.82 (2.01)	6.76 (1.79)	t-test	t = −3.13	0.002
**Routine execution**					
Door closed	13 / 85	30 / 30	χ2—test	χ2[1, *N* = 115] = 64.4	< 0.001
Used medical gloves	42 / 85	29 / 30	χ2—test	χ2[1, *N* = 115] = 18.2	< 0.001
Used hand disinfection	18 / 85	28 / 30	χ2—test	χ2[1, *N* = 115] = 45.1	< 0.001

The statistical comparisons of routine execution refer to the general routine execution (independent of the specific point in time)

## Discussion

Results showed that the levels of immersion differed significantly between the VR-based simulation and the actor-based simulation. This is confirmed by questionnaire data as well as the difference in the execution of medical routines. Specifically, the huge differences between routine execution in the two groups were striking. While both settings (VR vs. actor-based simulation) were able to achieve the primary learning objectives well, there were clear differences in the achievement of the secondary learning objectives related to medical routines.

### Differences in immersion

The obtained differences in immersion between the two types of simulated environments are not unexpected. While the actor-based simulations are well-optimized and have been an integral part of the curriculum for years, the VR-based simulation was a prototype with several shortcomings. Some aspects of the application (e.g. the visual quality of the light and digital surfaces, complex interactions like pushing aside the patient´s hair to examine the scalp) have in part been simplified due to technical and time constraints. Furthermore, it became clear that for some students, the technical barriers in using the system were higher than for others, which could have also deteriorated the immersion of the experience. In future VR software versions, tackling these shortcomings is expected to increase the immersion of the simulation. In general, VR software and hardware have undergone significant improvements in recent years. To reach a nearly perfect immersive experience as it is in real life simulations there needs to be further developments fulfilled.

Furthermore, VR technology in general currently has certain drawbacks that need to be overcome to create an experience that is almost indistinguishable from the real world. For example, the fixed focal distance, which makes it impossible to focus on close range due to the optical illusion created by the VR headset (“Vergence-Accommodation Conflict”, VAC) is something that can hinder an immersive experience [50]. More importantly, most current VR applications are focused on visual experiences, neglecting other components of a real-world multisensory perception. Future technical development may be able to integrate the users´ bodies into the VR environment, to increase presence and embodiment further.

The Valve Index VR system used here has the capability to estimate the users´ finger orientation with the individual digits being either extended or closed. The emergence of finger tracking technology [47, 48] for the consumer market may render this technique obsolete and offer newfound opportunities for interacting with the VR scenario. Alternatively, haptic gloves, which simulate hapto-tactile feedback whenever users interact with objects in the digital world, may further enhance the feeling of presence [49]. Even though optics and acoustics already work at a very high level, there is currently no commercial hardware that relies on olfactory stimuli. These are particularly relevant in a medical context [50] and can have a significant influence on behavior, as Birnbach et. al. could show [51].

To sum up, a high level of immersion comparable to the real world cannot be achieved with the currently available commercial hardware and the development possibilities of non-big players. However, the (rapid) development of new hardware and software in the VR sector [51] provides a promising outlook, which means that significant improvements in this area can be expected in the near future.

### Differences in primary and secondary learning objectives

To our knowledge, our study is the first to gather empirical data comparing primary and secondary learning objectives in a VR-based simulation in medical education. Our results showed a huge difference in the execution of medical routines between the two simulated environments. The fact that almost all students in the actor-based simulation exerted the expected behavior shows that the students are generally able to adhere to these basic behaviors to a very high degree without further prompting at this stage of their training. Thus, the lack of execution of medical routines in the VR group does not seem to be related to a lack of knowledge or competence. We propose that elements of the simulated environment, notably immersion and missing multisensory experience, in combination with the content of the medical routines may account for the observed results. One could expect that olfactory input (e.g. odour of perspiration or wounds) would increase the proportion of students wearing gloves in the VR-based simulation. At the same time, feeling human presence in front of the examination room and ambient noises would likely lead to a higher proportion of students closing the door of the examination room. These hypotheses should be examined in future studies and with the technological advances yet to come.

In summary, it was found that a moderate level of immersion is generally sufficient for training primary skills. However, current VR applications do not appear to fully support the training of secondary skills, such as the correct use of personal protective equipment or the appropriate performance of hand hygiene. For such skills, actor-based simulated patient training appears to be more appropriate.

### Performance as immersion indicator

We aimed to clarify if we can use medical routines as an alternative and indirect assessment that does not suffer from the shortcomings of questionnaire data. This is supported by our data, which show that medical routines are able to differentiate better between the two simulated environments (VR-based/actor-based) than questionnaire data.

This makes the assessment of the execution of medical routines an interesting and time-saving alternative for measuring immersion in medical simulations. Our findings indicate the potential for such an indirect measure to more effectively distinguish between non-immersive and immersive simulations. From a methodological perspective, a multi method approach of assessing a construct is always preferred over a single measurement method. However, it is necessary to examine whether the differences in the execution of medical routines in other applications are also a measure of immersion in the simulated environment, or whether other factors cause these differences. Furthermore, it would be interesting to investigate if the results could be replicated in other domains with other routines apart from the medical context.

### Limitations

In our study we identified learning objectives as an indirect measurement of immersion. These two items are related, because we would expect that an immersive environment leads to better learning of both primary and secondary objectives. The extent to which the two items are related and which other variables play a role here would have to be investigated in further studies.

Furthermore, we already claimed that the two simulations differ in certain main aspects (e.g., course timeline, time in the simulated environment, specific topic), therefore we have to interpret the results with caution. Regarding the execution of medical routines, we cannot rule out that the instructions of the lecturers differed, because both lectures were part of the curriculum and thus not a fully controlled experimental design.

To compare the two environments, we used a questionnaire originally designed for computer-based simulations (RJPQ) and used it with minor adjustments for the actor-based simulation. It would have been better to use a measure that is validated for both types of simulations, but to our knowledge such a measure does not exist yet. It must also be considered that the purchase and development of VR software incurs high costs, particularly in the short term. These can pay off in the long term if the hardware is well utilised and actor salaries are saved.

Because we studied medical students, we cannot readily generalize our results to other medical professions with different socio-demographic characteristics. It would be interesting to see if the obtained results would be replicated using e.g., future nurses or experienced physicians.

## Conclusion

To summarize, it can be said that VR can be a helpful addition for medical education in the future. Specialized software for HMD-based immersive VR can be used to train learning objectives that could previously only be presented theoretically or inadequately (complex emergency scenarios, rare illnesses, potentially dangerous situations etc.). These learning objectives also appear to be well addressable with a medium level of immersion. For this purpose, it is very important to select the field of application specifically in order to achieve added value compared to conventional methods (e.g. simulation patients, manikins etc.) using the technology. In contrast to this are secondary learning objectives such as basic hand hygiene activities and the protection of the patient's personal integrity. A much higher level of immersion seems to be necessary here, which is not achieved with currently used methods in VR simulation.

The measurement of these secondary/basic skills identified here also appears to be a good instrument for using a further objective measurement tool in addition to the established subjective questionnaires, which specifically reflects the suitability for train deeper learning objectives.

This knowledge can be used to plan courses in a targeted manner and expand the training of students in the best possible way.

## Supplementary Information

Supplementary Material 1.

Supplementary Material 2.

## Data Availability

The datasets used and analysed during the current study are available from the corresponding author on reasonable request.

## Abbreviations

AR: Augmented reality
COVID: Coronavirus disease
e.g.: Exempli gratia / for example
HMD: Head-Mounted-Display
m: Minute
MR: Mixed reality
NKLM: Nationaler Kompetenzbasierter Lernzielkatalog Medizin / eng. National Competence Based Learning Objectives Catalogue Medicine
RJPQ: Reality Judgement and Presence Questionnaire
s: Second
SUS: System Usability Scale
VR: Virtual reality
