# Supplementary figures and images for: Immersive learning in medical education: analyzing behavioral insights to shape the future of VR-based courses
Source: BMC Med Educ. 2024 Dec 3;24:1413. doi: 10.1186/s12909-024-06337-7 (PMC11616111; doi:10.1186/s12909-024-06337-7)

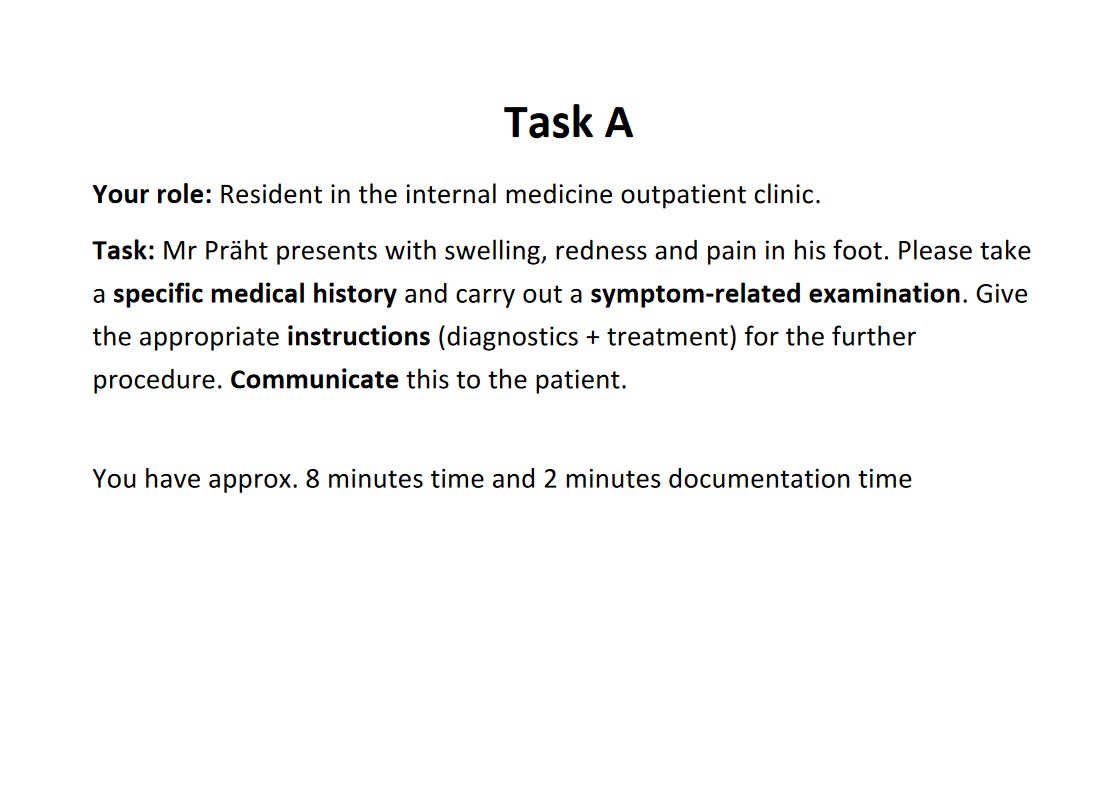

Supplement: Supplementary file 1 — Supplementary Material 1. [file 12909_2024_6337_MOESM1_ESM.jpg]
