# Supplementary material for: Immersive learning in medical education: analyzing behavioral insights to shape the future of VR-based courses
Source: BMC Med Educ. 2024 Dec 3;24:1413. doi: 10.1186/s12909-024-06337-7 (PMC11616111; doi:10.1186/s12909-024-06337-7)
Supplement: Supplementary file 2 — Supplementary Material 2. [file 12909_2024_6337_MOESM2_ESM.pdf]

medical training

You are a student in your practical year at a dermatology practice. Your supervisor was about to start a skin cancer screening, but received an important call at short notice.

**Please carry out a complete skin cancer screening.** You can enlarge your findings using the flatscreen in the room and save them for documentation.

The patient is already waiting in examination room 2. Your supervisor has left you a note with the patient's details, further information about the patient is not yet available.

*Patient: Müller, Udo*

*Date of birth: 18.10.1971*

*Initial assessment*

The findings will be discussed. Inside of the application it is possible to document skin lesions via images. These will be made available to you for the debriefing. Think carefully about which relevant clinical findings you would like to save. A maximum of **5 images** can be saved. The findings will be discussed in the plenary session.
